# Single Institution Experience in the Management of Locally Advanced (pT4) Differentiated Thyroid Carcinomas

**DOI:** 10.1245/s10434-024-15356-z

**Published:** 2024-05-03

**Authors:** Luca Sessa, Carmela De Crea, Nikolaos Voloudakis, Francesco Pennestri’, Luca Revelli, Pierpaolo Gallucci, Germano Perotti, Luca Tagliaferri, Ernesto Rossi, Esther Diana Rossi, Alfredo Pontecorvi, Rocco Bellantone, Marco Raffaelli

**Affiliations:** 1 Division of Endocrine and Obesity Surgery, Fondazione Istituto G. Giglio, Cefalù, Italy; 2 Division of Endocrine Surgery, Fatebenefratelli Isola Tiberina - Gemelli Isola, Rome, Italy; 3 Centro di Ricerca in Chirurgia delle Ghiandole Endocrine e dell’Obesità, Università Cattolica del Sacro Cuore, Rome, Italy; 4 Division of Endocrine and Metabolic Surgery, Fondazione Policlinico Universitario “Agostino Gemelli” - IRCCS, Rome, Italy; 5 Division of Nuclear Medicine, Fondazione Policlinico Universitario “Agostino Gemelli” - IRCCS, Rome, Italy; 6 Department of Diagnostic Imaging, Oncological Radiotherapy and Hematology - Gemelli ART (Advanced Radiation Therapy), Interventional Oncology Center (IOC), Fondazione Policlinico Universitario “Agostino Gemelli” – IRCCS, Rome, Italy; 7 Department of Medical Oncology, Fondazione Policlinico Universitario “Agostino Gemelli” – IRCCS, Rome, Italy; 8 Division of Anatomic Pathology and Histology, Fondazione Policlinico Universitario “Agostino Gemelli” - IRCCS, Rome, Italy; 9 Division of Endocrinology, Fondazione Policlinico Universitario “Agostino Gemelli” - IRCCS, Rome, Italy

## Abstract

**Background:**

Locally infiltrating (T4) differentiated thyroid carcinomas (DTC) represent a challenge. Surgical strategy and adjuvant therapy should be planned balancing morbidity and oncologic outcome. A series of patients with T4 DTC who underwent multidisciplinary evaluation and treatment is reported. The main study endpoints were the oncologic outcome, complication rates, and risk factors for tumor recurrence.

**Patients and Methods:**

All DTC cases operated between 2009 and 2021 were reviewed and T4 DTC cases were identified. En bloc resection of inferior laryngeal nerve (ILN), tracheal, and/or internal jugular vein (IJV) was performed in cases of massive infiltration. In cases of pharyngoesophageal junction (PEJ) invasion, the shaving technique was always applied.

**Results:**

Among 4775 DTC cases, 60 were T4. ILN infiltration was documented in 45 cases (en bloc resection in 9), tracheal infiltration in 14 (tracheal resection in 2), PEJ invasion in 11 (R0 resection in 7 cases and < 1 cm residual tissue in 4 cases), IJV resection in 6, and laryngeal in 2. In total, 11 postoperative ILN palsy, 23 transient hypoparathyroidisms, and 2 hematomas requiring reoperation were registered. Final histology showed 7 pN0, 22 pN1a, and 31 pN1b tumors. Aggressive variants were observed in 47 patients. All but 1 patient underwent radioiodine treatment, 12 underwent adjuvant external beam radiation therapy (EBRT), and 2 underwent chemotherapy. At a median follow-up of 58 months, no tumor-related death was registered, and seven patients required reoperation for recurrence. Tracheal invasion was the only significant factor negatively impacting recurrence (*p* = 0.045).

**Conclusions:**

A multidisciplinary approach is essential for the management of T4 DTC. Individualized and balanced surgical strategy and adjuvant treatments, in particular EBRT, ensure control of locally advanced disease with acceptable morbidity.

The increased thyroid cancer incidence-based mortality registered in the last decade suggests an increase in thyroid carcinoma incidence not completely accounted for by over-diagnosis of small indolent tumors.^1,2^

This is probably owing to a growing incidence of malignant tumors with aggressive features and at an advanced stage at presentation.^1,2^

A standard definition for advanced thyroid cancer does not exist, as it could be referred to as locally advanced disease/local invasion (strap muscles, inferior laryngeal nerve -ILN-, trachea, larynx, esophagus, vascular bundle, etc.), advanced regional disease (N1a/N1b disease), tumors with distant metastases, or in the presence of persistent/recurrent disease as consequence of aggressive disease and/or previous incomplete resection for local invasion.^1–6^

Independently from histological subtypes, stage T4 nodal involvement and/or distant metastatic disease for thyroid carcinoma includes gross extrathyroidal extension into major neck structures.^7–10^

On the other hand, both available treatments options and prognosis are strictly correlated to tumor biology since tumor staging for T4 advanced thyroid carcinomas varies from stage I (98–100% 10-year expected disease specific survival) for patients with follicular-cell derived differentiated thyroid carcinoma (DTC) < 55 years T4N1bM1 to stage IV for any patient with anaplastic thyroid carcinoma (5-year survival rate 7%).^7–10^

Surgery, radioiodine (^131^I) ablation (RAI), external beam radiation therapy (EBRT), and systemic treatment represent the available resources that could be evaluated by multidisciplinary teams (surgeon, endocrinologist, pathologist, radiologist, nuclear medicine physician, radiotherapist, oncologist, and anesthesiologist) obtaining a comprehensive assessment.^1,2,11–32^

In this setting, surgical strategy and other available treatments should be planned balancing morbidity associated with surgical resection, patients’ quality of life and local control of the disease, risk of persistence/recurrence, and survival outcome benefit.^3,4^

Moreover, many experiences in the treatment of advanced thyroid tumors reported discordant results, probably because of study population bias related both to initially resectable/unresectable tumors and to different tumor biology (i.e., follicular cell-derived cancer, anaplastic thyroid carcinoma, and medullary thyroid carcinoma).^11–19^

We aimed to report operative and oncologic results of a series of patients with T4 DTC who underwent surgery with curative intent and integrated treatment strategy at a single institution.

## Patients and Methods

### Study Design

All DTC cases operated between January 2009 and December 2021 were reviewed and T4 DTC cases were identified. For each included patient, age, sex, tumor size, date of surgery, operative time, previous thyroid operation, extension of thyroidectomy, type and extension of lymph node dissection (central neck dissection, lateral neck dissection, unilateral, or bilateral), evidence and type of gross extrathyroidal extension (i.e., larynx, trachea, esophagus, ILN, etc.), tumor resection margins (R0, R1, R2), evidence of postoperative complications (hypoparathyroidism, ILN palsy, neck hematoma, and lymphatic leaks), hospital stay, and pathological TNM classification were registered in a specifically designed deidentified database.

The current study was approved by the ethics committee of “Fondazione Policlinico Universitario Agostino Gemelli—IRCCS”, Rome (Protocol ID 4999, approval number 0017844/22). The study has been registered on clinicaltrial.gov (PRS review pending).

### Preoperative Assessment

Following physical examination and study of the biochemical profile, all patients underwent ultrasound evaluation and fine needle aspiration biopsy (FNAB). All aspirations (usually with two passes performed for each thyroid lesion) were performed with 25–27 G needles. No rapid on-site assessment for adequacy of material was performed. All patients consented to this procedure. All FNAC specimens were processed using a ThinPrep 5000TM processor (Hologic Co., Marlborough, MA, USA). Prepared slides were fixed in 95% methanol and stained with a Papanicolaou stain. Any remaining material was stored in Preservcyt solution for potential ancillary studies. Core-biopsy was performed in selected cases to exclude dedifferentiated tumors and tumors not arising from follicular thyroid cells (i.e., thyroid lymphoma and metastatic tumors).

All the patients underwent flexible laryngoscopy assessing the preoperative status of vocal cord motility.

Computed tomography (CT) and/or magnetic resonance imaging (MRI) were used to evaluate extension of the disease, soft tissue/muscular, vascular and/or laryngeal/tracheal and/or visceral invasions.

^131^I whole body scan and 18-fluorodeoxyglucose positron emission tomography (18F-FDG PET) were selectively proposed especially in tumor recurrences.

Tracheoscopy, bronchoscopy, and/or esophagoscopy were used in cases of suspected infiltration of the trachea and/or esophagus.

After the initial workup, a comprehensive assessment of the case and indications for subsequent treatment were developed by an institutional multidisciplinary thyroid cancer tumor board.

### Surgical Approach

All the surgical procedures were performed by an experienced endocrine surgeon. In selected cases thoracic surgeons, vascular surgeons and/or otolaryngologists were involved to obtain a multispecialist approach during the same operation. Intermittent intraoperative nerve monitoring was used in all the procedures.

In cases judged as resectable T4 DTC, every effort was made to obtain an en bloc resection with R0 tumor margins following the principles of fascial dissection.^33,34^

To ensure local disease control, intraoperative confirmation of resection margins was obtained in most cases with frozen section examination (i.e., malignant tissue infiltrating neck structures).

The sternothyroid muscles were resected en bloc with their fascial layers and tumor in all cases. More extended resections, including the sternohyoid and omohyoid muscles and, exceptionally, the sternocleidomastoid muscle was performed only in cases of overt involvement and/or infiltration.

In cases of ILN invasion, every effort was made to preserve its anatomical and functional integrity if it was functioning normally preoperatively; en bloc resection of the cervical tract of ILN was performed in cases of preoperative evidence of vocal cord paralysis or in cases where en bloc resection was demanded for R0 resection (in the absence of other sites of gross tumor invasion).

Tracheal and/or laryngeal resections were performed to obtain R0 resection only in cases of intraluminal spread, where disease free and overall survival benefits outweighed the potential surgical morbidity, as proposed by the multidisciplinary team consultation. In similar cases, patients were extensively informed and counseled before the operation and their willingness taken into account.

Unilateral internal jugular vein (IJV) resection was performed in cases of gross extrathyroidal extension of tumor or extranodal extension of involved lymph nodes.

In cases of esophagus and/or pharyngoesophageal junction (PEJ) invasion, shaving technique was always applied.

Titanium clips were used liberally to delimitate the site of infiltration with positive margins (potential R1 or R2 resections) eventually facilitating EBRT.

All surgical pathology specimens were fixed in 10% buffered formaldehyde, embedded in paraffin and 5 micron-thick sections then stained with hematoxylin and eosin (H&E).

Pathologic tumor staging was defined in accordance with the 2017 eighth edition of the American Joint Committee on Cancer pTNM staging system.^7^ In cases of patients operated on before 2017, the specimens were reviewed and reclassified by an expert pathologist in accordance with the 2017 eighth edition of the AJCC pTNM staging system.

### Postoperative Evaluation

The protocol for the postoperative management of hypoparathyroidism has been previously described.^35^ Fiberoptic laryngeal evaluation was performed in all the cases confirming preoperative vocal cord paralysis or evaluating postoperative ILN palsy.

All patients received levothyroxine (LT4) suppressive treatment. All patients were classified as high risk in the American Thyroid Association (ATA) risk score^5^ and were offered RAI according to the ATA Guidelines. The median administered activity was 5.55 GBq (range 3.7–7.4 GBq). We did not perform individual dosimetry, but we used adjusted/fixed activities. Thyroid-stimulating hormone (TSH)-stimulated sTg levels obtained prior to RAI and posttherapy whole body scans (TxWBS) were evaluated. Patients with TxWBS that revealed no thyroid remnants or metastases were followed up with neck ultrasound and serum thyroglobulin (sTg). Where TxWBS revealed thyroid remnants or metastases, these patients underwent new DxWBS and sTg off LT4 3 months after the previous RAI or underwent new treatment after 6 months.

In cases of R1–R2 resections, distant metastases, and/or unresectable recurrence following RAI, patients were evaluated for further treatment with RAI or EBRT and/or systemic treatment.

Regarding EBRT, a conventional fractionation was used, and the prescription dose was 66 Gy targeted at the tumor bed of patients with no evidence of residual disease and 70 Gy for patients with evidence of residual disease on the functional imaging. Volumetric modulated arc therapy (VMAT) was performed. Adverse events were monitored according to the Common Terminology Criteria for Adverse Events (CTCAE) v4.0.

Systemic therapy was established considering the histological features of residual tumor. In case of aggressive variants, a combination regimen with carboplatin AUC 5 and paclitaxel 175 mg/m^2^ every 21 days was employed.^36^

Follow-up data were obtained by outpatient consultations or telephone contact.

### Definitions

Total thyroidectomy was defined as total, bilateral extracapsular thyroid removal.

Unilateral central neck dissection included prelaryngeal, pretracheal, and the paratracheal nodes on the side of the tumor (paratracheal nodes contralateral to the tumor were not included).^37^ Bilateral central neck dissection included the removal of prelaryngeal, pretracheal, and both the left and right paratracheal nodes.^37^ Lateral neck dissection was performed in all the cases with therapeutic intent. Comprehensive lateral neck dissection was defined as compartment oriented functional lateral neck dissection, including levels II, III, IV, and V^38^.

Resection margins were considered free (R0) if frozen section analysis (confirmed by definitive analysis) showed no tumor in the soft tissue or cartilaginous margins. Margins were considered microscopically invaded (R1) if definitive analysis showed tumor at the margin of the operating specimen or < 1 mm from the margin.^39^ Resection margins were considered R2 in cases of macroscopic evidence of residual disease.

Local recurrent disease was defined as clinically detectable disease in the thyroid bed. Nodal recurrent disease was defined as clinically detectable disease in the central and/or lateral compartment lymph nodes. Distant metastases (disease outside the thyroid bed and cervical lymph node) were identified in the presence of an elevated sTg and/or sites of uptake on postoperative radioactive iodine scan, eventually confirmed by imaging studies.

### Study Endpoints

The primary endpoint was to evaluate the effectiveness of integrated surgical and medical approach on T4 DTC in terms of oncologic outcome.

The secondary endpoint was to assess the complication rate in patients who underwent surgery for T4 DTC and to assess risk factors for tumor recurrence.

### Statistical Analysis

Distribution of variables was assessed with the Shapiro-Wilk test. Continuous variables were reported as median (range, minimum-maximum value, or interquartile range - IQR) owing to nonparametric distribution. Differences between groups were assessed by the Mann–Whitney U test. Categorical variables were analyzed using chi-square and Fisher’s exact test, as appropriate. Survival was estimated with the Kaplan–Meier method and compared with the log-rank test. *P* values less than 0.05 were considered statistically significant. All analyses were two-tailed. Data analysis was performed with IBM SPSS Statistics for Windows, Version 25.0 (Armonk, NY: IBM Corp).

## Results

Demographic, clinical, operative, postoperative, pathologic, and follow-up characteristics of all the included patients are reported in Table 1.

**Table 1:** Demographic, operative, and histologic characteristics of included patients

	Population characteristics
Number	60
Age (y)	
Median and IQR	57 (43.5–68.5)
Sex (M/F)	24 (40%)/36 (60%)
Previous operation (y)	10 (16.7%)
Tumor size (mm)	
Median and IQR	21 (13–38.5)
Extent of thyroidectomy^a^	
N/A	8 (13.3%)
TT	50 (83.3%)
Completion Thyroidectomy	2 (3.4%)
Extent of CND^b^	
Not performed	7 (11.7%)
Unilateral	7 (11.7%)
Bilateral	46 (76.7%)
Extent of LND^c^	
Not performed	29 (48.3%)
Unilateral	24 (40%)
Bilateral	7 (11.7%)
Infiltration pattern (y)	
Inferior laryngeal nerve	45 (75%)
Trachea	14 (23.3%)
Pharyngoesophageal	11 (18.3%)
Internal Jugular vein	6 (10%)
Larynx	2 (3.3%)
Operative time (min)	
Median and IQR	200 (112.5–300)
Resection margin status	
R0	47 (78.3%)
R1	9 (15%)
R2	4 (6.7%)
N status	
No	7 (11.7%)
N1a	22 (36.7%)
N1b	31 (51.7%)
Aggressive histology (presence)	47 (78.4%)
Distant metastasis (presence)	2 (3.4%)

^a^*TT* total thyroidectomy

^b^CND central neck dissection

^c^*LND* lateral neck dissection

### Patient Population

Among 4775 DTC cases, 60 (1.25%) were pT4a at final histology. There were 24 males (40%) and 36 females (60%) with a median age of 57 years. Overall, 50 patients (83.3%) underwent total thyroidectomy, 10 patients (16.7%) were referred to our center for recurrence after undergoing a primary operation in other centers with final histology showing pT4 primary tumors: in eight cases (13.3%) no thyroid resection was performed (previous total thyroidectomy) and two patients (3.4%) underwent completion thyroidectomy (previous partial thyroidectomy).

Preoperative work-up showed vocal cord paralysis in eight (13.3%) cases, definite tracheal invasion in seven (11.7%) cases, laryngeal invasion in one patient (1.7%), IJV infiltration and/or thrombosis in six (10%) patients. All the patients were scheduled for surgery after comprehensive multidisciplinary tumor board evaluation.

### Surgical Approach

All patients were operated with the cervical approach, and in one case (1.7%) concomitant sternotomy was accomplished. Overall, ILN infiltration was documented in 45 cases (75%; en bloc resection in 9 cases), tracheal infiltration in 14 (23.3%; tracheal resection in 2), PEJ invasion in 11 (18.3%; R0 resection in 7 cases and < 1 cm residual tissue in 4 cases), IJV resection in 6 (10%), and laryngeal in 2 (3.3%; both underwent “shaving” resection).

### Postoperative Evaluation

Postoperative outcomes, adjuvant treatments and follow-up data are reported in Table 2. Overall, 20 (33.3%) postoperative ILN palsy cases were registered, including 9 patients who underwent en bloc ILN resection; 14 (23.3%) of these cases resulted permanent vocal fold paralysis.

**Table 2:** Postoperative outcomes, adjuvant treatment and follow-up

	Number of patients
Early complications	
VFP^a^	20 (33.3%)
Hypoparathyroidism	23 (38.3%)
Reoperation for hematoma	2 (3.4%)
Lymphatic leak	2 (3.4%)
Other	1 (1.7%)
Late complications	
Permanent VFP	14 (23.3%)
Permanent hypoparathyroidism	9 (15%)
Other	1 (1.7%)
Postoperative stay (days)	
Median and IQR	3 (2–4)
Adjuvant treatment	
RAI^c^	59 (98.3%)
EBRT^d^	12 (19.4%)
Chemotherapy	2 (3.4%)
Follow-up time^b^ (months)	
Median and range	58 (11–126)
Recurrence	7 (11.7%)

^a^*VFP* vocal fold paralysis, includes VFP owing to nerve resection (9 cases) and excessive surgical manipulation/shave resection (11 cases), there were 2 bilateral cases (both permanent on one side, and transient on the other)

^b^Three patients lost to follow-up at 24, 39, and 46 months following operation

^c^*RAI* radioactive iodine

^d^*EBRT* external beam radiation therapy

In total, 23 (38.3%) transient hypoparathyroidisms, two hematomas (3.4%), and one lymphatic leak (1.7%) required reoperation.

Final histology showed 7 (11.7%) pN0, 22 (36.7%) pN1a, and 31 (51.7%) pN1b tumors. Aggressive variants were observed in 47 cases (78%). In total, 29 patients presented with tall cell, 11 with columnar, 3 with solid, 2 with hobnail, and 2 with diffuse sclerosing variants.

### Postoperative Treatments and Follow-up

All but one patient (denied therapy) underwent RAI treatment, 12 (19.4%) underwent adjuvant EBRT, and 2 (3.4%) underwent chemotherapy with carboplatin + paclitaxel.

The median time from surgical intervention to the start of the adjuvant EBRT was 5 months (range 2–6). Skin atrophy, oral mucositis, and dysphagia were the most common reported side effects with 60% grade 1 and 40% grade 2. No grade 3 or higher toxicity were recorded.

At a median follow-up of 58 (range, 11–126) months, no tumor-related death was registered. Overall, seven patients (11.7%) required reoperation for recurrence, six patients were reoperated for lymph node recurrence, and one for recurrence in the pharynx/retropharyngeal space. It should be noted that EBRT as an adjuvant treatment was effective in the majority of patients with R1/R2 disease, since only one patient developed a local recurrence. In addition, the two patients that underwent formal tracheal resection, did not present a recurrence thus far.

**Fig. 1.**
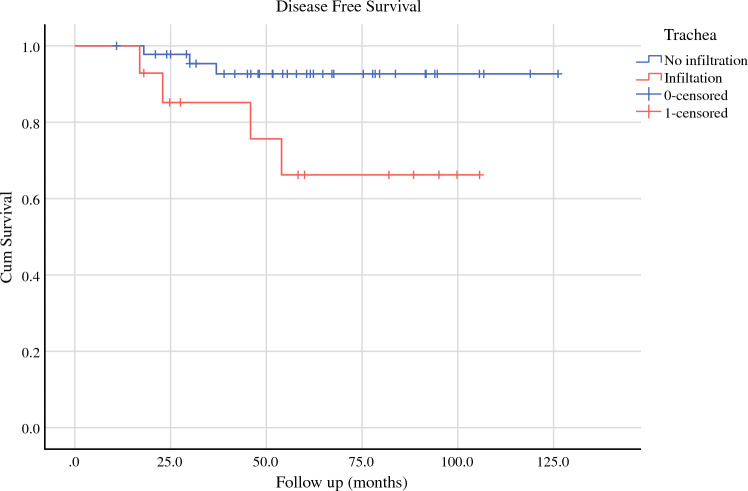
Disease free survival between patients with and without tracheal invasion. For no tracheal infiltration the mean DFS: 119.1 months (95% CI 111.3–126.8) versus 82.5 months (95% CI 63.4–101.5), *p* = 0.023

At univariate analysis (Table 3), the only significant factor for recurrence was tracheal involvement (*p* = 0.045). The mean disease-free survival (DSF) was significantly different between patients with tracheal involvement and those without [mean DFS: 119.1 months, 95% confidence interval (CI): 111.3–126.8) versus DSF: 82.5 months, CI: 63.4–101.5, *p* = 0.023; Fig. 1]. “R *status*” and local invasion management did not yield significant differences for recurrence.

**Table 3:** Comparative analysis between recurrence and no recurrence groups

	No recurrence	Recurrence	*p*
Number	53	7	
Age (years)			
Median and IQR	56 (40.5–67.75)	62 (48–74)	0.27
Sex (M)	20 (37.7%)	4 (57.1%)	0.422
Previous operation	8 (15.1%)	2 (28.6%)	0.330
Tumor size (mm)			
Median and IQR	20 (12–38.5)	30 (20.75–51)	0.141
Infiltration pattern			
Inferior laryngeal nerve	42 (79.2%)	3 (42.9%)	0.058
Trachea	10 (18.9%)	4 (57.1%)	**0.045**
Pharyngoesophageal	8 (15.1%)	3 (42.9%)	0.108
Internal jugular vein	5 (9.4%)	1 (14.3%)	0.541
Larynx	2 (3.8%)	0 (0%)	1
Infiltration management			
Inferior laryngeal nerve (formal resection/shaving)	7 (16.7%)/35 (83.3%)	2 (66.6%)/1 (33.3%)	0.105
Trachea (formal resection/shaving)	2 (20%)/8 (80%)	0 / 4 (100%)	0.330
Pharyngoesophageal (no remnant/remnant)	5 (62.5%)/3 (37.5%)	2 (66.6%) / 1 (33.3%)	0.108
Larynx (formal resection/shaving)	0/2 (100%)	0 / 0	0.137
Multiple site infiltration	15 (28.3%)	4 (57.1%)	0.193
Resection margin status			0.587
R0	42 (79.2%)	5 (71.4%)	
R1	8 (15.1%)	1 (14.3%)	
R2	3 (5.7%)	1 (14.3%)	
N status			0.889
No/Nx	6 (11.7%)	1(14.3%)	
N1a	20 (37.7%)	2 (28.6%)	
N1b	27 (50.6%)	4 (57.1%)	
Adjuvant treatment			
RAI^a^	52 (98.1%)	7 (100%)	1
EBRT^b^	11 (20.8%)	1 (14.3%)	1
Chemotherapy	2 (3.8%)	0 (0%)	1
Aggressive histology (presence)	41 (77.4%)	6 (85.7%)	0.614
Distant metastasis (presence)	1 (1.9%)	1 (14.3%)	0.222

^a^*RAI* radioactive iodine

^b^*EBRT* external beam radiation therapy

## Discussion

Results of the present study confirm the effectiveness of multidisciplinary approaches to locally advanced DTC. In particular, surgical strategies modulated on patients’ characteristics seem adequate to ensure good results in terms of local control of disease, risk of complications, and good quality of life, avoiding excessive mutilation surgery. EBRT, accompanied with RAI is of utmost importance to improve disease free recurrence rate, especially in patients with non-R0 margins after surgical resection. Results obtained in centers where multidisciplinary approaches and multimodal integrated therapies are available confirm the importance of referring complex cases of thyroid cancers to referral centers.

Surgery, RAI, EBRT, and systemic therapy represent the available resources that could be evaluated by multidisciplinary teams to plan the tailored treatment strategy for each patient with malignant thyroid tumors with aggressive features and advanced disease.^1,2,11–32^

A comprehensive assessment involving endocrine surgeons, head and neck surgeons, thoracic surgeons, and vascular surgeons, endocrinologists, pathologists, radiologists, nuclear medicine physicians, radiotherapists, oncologists, and anesthesiologists is essential to ensure an adequate individualized treatment.^3,4,11–19^

Advanced thyroid cancer should not be an unexpected intraoperative finding, since initial surgery plays a crucial role. In this setting, preoperative work up is essential to define the biological aggressiveness of the tumor, invaded anatomic structure/s, primary tumor invasion/nodal disease invasion, extension of disease (limited to central neck, extended to lateral neck, extended to mediastinum, and distant metastases).^3,4,11–19,40–42^

Evaluation of patient’s characteristics and physical examination, biochemical and genetic profile, neck ultrasound with FNAB and/or core biopsy, CT/MRI, 18F-FDG PET, laryngeal evaluation, tracheoscopy, bronchoscopy, and esophagoscopy are the available tools for an optimal initial staging, which will guide further treatments.^3,4,11–19,40–42^

Firstly, an adequate preoperative pathology study is essential to distinguish advanced thyroid carcinomas from lymphomas and metastasis, while, in cases of advanced thyroid cancer, aggressive DTC from medullary thyroid carcinoma and anaplastic thyroid carcinoma.^3,4,10,43,44^

Secondly, preoperative studies are paramount to assess the maximum possible benefit obtainable by aggressive surgery (gross extrathyroidal extension invading subcutaneous soft tissues, larynx, trachea, esophagus, inferior laryngeal nerve—T4a—versus gross extrathyroidal extension invading prevertebral fascia or encasing carotid artery or mediastinal vessels—T4b).^3,4,7–9^

In case of unresectable tumors, neoadjuvant therapy and/or palliative treatments are proposed; and the patients are referred for surgery if a restaging work up demonstrates the possibility of a surgical approach realistically improving oncologic outcome.^30–32^

In case of resectable tumors, the surgical strategy is planned considering extension of the disease (cervicotomy versus cervicotomy and sternotomy^19^), the necessity of other specialists during the operation (i.e., thoracic surgeon, vascular surgeon), and intraoperative adjuncts (i.e., intraoperative neuro monitoring, near-infrared-induced autofluorescence/indocyanine green angiography for parathyroid identification/preservation, energy-based devices, prosthetic materials, etc.).^14,45,46^

Surgical approach should be both pre- and intraoperatively modulated balancing postoperative morbidity of surgical mutilation, quality of life, and oncologic benefit in light of other available adjuvant treatments.^1–4,11–32^

After surgery, a subsequent multidisciplinary team evaluation is essential to plan further treatment modalities, taking into account the surgical resection margins achieved (R0, R1, R2), the postoperative course (type of complications versus uneventful), and the final histology.^1–4,11–32^

In this study, resection margin status did not affect disease-free survival when surgery was accompanied by appropriate adjuvant treatment. It is also worth pointing out, that in our series only tracheal involvement had a significant negative impact on DFS, while ILN involvement exhibited a strong trend for favorable outcomes. Those findings are in accordance with the majority of published literature on locally advanced DTC.^47,48^

However, discordancy in outcomes of patients with advanced thyroid tumors, as reported in the pertinent literature, may be attributed to selection bias of the study population, since cases included exhibited variations both in tumor biology and in resectability criteria.^11–19^

In the present study, the operative and oncologic results of a series of T4 DTC who underwent surgery with curative intent and integrated treatment strategy at a single institution are reported.

Interestingly, concerning recurrence, only one of seven cases had a recurrence on the site of local infiltration, while the rest concerned regional lymph nodes. In a publication by Chan et al.,^49^ it was reported that local recurrence might lead to secondary operations, requiring extensive resections and reconstructions followed by high morbidity. Such an event was not observed in our series, as in the second operation of this case, local control was achieved with a conservative “shave” approach. One could also argue that the low rate of local recurrences in the present series could be attributed to the liberal and potentially effective use of EBRT in patients with suspected R1 or R2 resection.

However, it has to be underlined that the present study has some limitations. First, it is a retrospective study and lacks an overall cohort analysis of all patients presenting with cT4 DTC during the same study period. As such, oncologic outcomes and disease progression sequalae of nonoperated patients were not reported. On the other hand, the study was designed to evaluate the effectiveness of an integrated surgical and medical approach on T4 DTC in terms of oncologic outcome, assessing the complication rates and potential risk factors for tumor recurrence in patients who underwent surgery for T4 DTC.

In addition, the absence of longer follow up data probably does not ensure adequate information regarding long term outcomes essential when dealing with thyroid carcinoma patients, while the relatively small number of recurrences might have not enabled us to exhibit all potential risk factors.

Nonetheless, one of the strong points of the present study is that the results showed the impact of multidisciplinary treatment of a complex disease in a referral center in a relatively short period using the same multidisciplinary mentality both for surgical strategies and adjuvant therapy.

In conclusion, a multidisciplinary approach is essential for the management of T4 DTC. An individualized and balanced surgical strategy and adjuvant treatments, in particular EBRT, ensure control of locally advanced disease with acceptable morbidity.
